# Evaluation of the additional occupational risk for workers with orthopedic implants in MRI settings

**DOI:** 10.1093/annweh/wxag067

**Published:** 2026-08-26

**Authors:** Luca Zilberti, Alessandro Arduino, Simone Busoni, Vincenzo Camisa, Vittorio Cannatà, Andrea Magistrelli, Vittorio Miele, Nadia Oberhofer, Daniela Origgi, Silvia Pradella, Umberto Zanovello, Oriano Bottauscio

**Affiliations:** Istituto Nazionale di Ricerca Metrologica, Divisione Metrologia dei materiali innovativi e scienze della vita, - Settore Campi e sistemi elettromagnetici, Strada delle Cacce 91, Torino 10135, Italy; Istituto Nazionale di Ricerca Metrologica, Divisione Metrologia dei materiali innovativi e scienze della vita, - Settore Campi e sistemi elettromagnetici, Strada delle Cacce 91, Torino 10135, Italy; Health Physics Department, Azienda Ospedaliero-Universitaria Careggi, Largo Brambilla 3, Firenze 50134, Italy; Unità Operativa Complessa (UOC) Medicina del Lavoro—IRCCS Ospedale Pediatrico Bambino Gesù, Piazza S. Onofrio 4, Roma 00165, Italy; Unità Operativa Complessa (UOC) Fisica Sanitaria—IRCCS Ospedale Pediatrico Bambino Gesù, Piazza S. Onofrio 4, Roma 00165, Italy; Diagnostic Radiology Unit, IRCCS Ospedale Pediatrico Bambino Gesù, Palidoro, Via della Torre di Palidoro, Roma 00054, Italy; Department of Emergency Radiology, Azienda Ospedaliero-Universitaria Careggi, Largo Brambilla 3, Florence 50134, Italy; Department of Experimental and Clinical Biomedical Sciences “Mario Serio”, University of Florence, P.zza S. Marco, Firenze 50121, Italy; Servizio Aziendale di Fisica Sanitaria, Azienda Sanitaria Alto Adige SABES, Via L. Böhler 5, Bolzano 39100, Italy; Istituto Europeo di Oncologia, IRCCS, Via Ripamonti 435, Milano 20141, Italy; Department of Emergency Radiology, Azienda Ospedaliero-Universitaria Careggi, Largo Brambilla 3, Florence 50134, Italy; Istituto Nazionale di Ricerca Metrologica, Divisione Metrologia dei materiali innovativi e scienze della vita, - Settore Campi e sistemi elettromagnetici, Strada delle Cacce 91, Torino 10135, Italy; Istituto Nazionale di Ricerca Metrologica, Divisione Metrologia dei materiali innovativi e scienze della vita, - Settore Campi e sistemi elettromagnetici, Strada delle Cacce 91, Torino 10135, Italy

**Keywords:** electromagnetic fields, magnetic resonance imaging, occupational exposure, orthopedic implants, safety

## Abstract

**Objective:**

To provide quantitative information that can aid the relevant authority in deciding whether workers with metallic nonferromagnetic orthopedic implants can work in proximity to magnetic resonance imaging (MRI) scanners, including when the exam is being performed.

**Methods:**

Virtual experiments in which the exposure of workers with and without prostheses is evaluated through numerical simulation of realistic human models, with reference to 4 physical effects: radiofrequency (RF)-induced heating, gradient-induced heating, gradient-induced peripheral nerve stimulation, and mechanical actions related to the Lenz effect.

**Results:**

In the presence of the considered implants, the extent of RF-induced heating and of the electric field responsible for peripheral nerve stimulation is low and comparable to what is observed without the prostheses. The extent of gradient-induced heating and Lenz effect, which occur only in the presence of implants, is minimal.

**Conclusion:**

As long as the body of the worker with passive non-ferromagnetic orthopedic implants remains outside the MRI scanner, the incremental risk posed by the presence of the implants is negligible. The Lenz effect is weak even if the part of the worker's body with an implant enters the scanner bore.

What's Important About This Paper For personnel performing magnetic resonance imaging (MRI), a competent authority is required to certify fitness for the job, which requires consideration of orthopedic implants. This study found that workers with nonferromagnetic passive implants exposed to MRI electromagnetic fields will experience radiofrequency heating and nerve stimulation similar to those without implants, while gradient-related heating and mechanical effects are minimal. Overall, incremental risk from passive implants is negligible outside the scanner.

## Introduction

With an estimated volume of 150–200 million scans performed worldwide each year (How Many Medical Imaging Scans 2025), magnetic resonance imaging (MRI) is one of the most prevalent medical imaging modalities. The use of MRI involves different professionals who may work in close proximity to the scanners, including radiologists, medical physicists, radiographers, nurses, anesthetists, technicians, engineers, and cleaners (Hansson Mild et al. 2013). These workers may be exposed to the stationary magnetic field of the scanner (which, in most cases, is always active) and, less frequently, to the time-varying switched gradient and radiofrequency fields (which are generated during the scans). Due to the presence of these fields, the use of MRI scanners entails particular hazards, the foremost being the “projectile effect,” which involves ferromagnetic objects being violently attracted toward the scanner. Because of these risks, it is mandatory that all MRI workers undergo MRI safety training.

In Europe, the main reference regulating workers' exposure to electromagnetic fields is Directive 2013/35/EU (European Directive 2013/35/EU). According to the Directive, the employers shall assess the risk to the workers. During the assessment, special attention shall be paid to “workers at particular risk”, including those who wear implanted medical devices. The Directive acknowledges that its requirements may conflict with certain activities, including MRI, for which a derogation is provided. This derogation can be granted if the employer demonstrates that the workers are still protected against safety risks. In Member States, the requirements provided by the Directive have to be incorporated into national laws. In the country of the present authors (Italy), workplace safety is regulated by Decree 81/2008 (Italian Legislative Decree n. 81, 2008) and its later amendments. More specifically, the implementation of Directive 2013/35/EU took place through the Legislative Decree 159/2016 (Italian Legislative Decree n. 159, 2016), corroborated by a decree of the Ministry of Health issued on 14 January 2021 (Decree of the Italian Ministry of Health 2021). According to these laws, a specific professional figure (“medico competente”, ie a physician specialized in occupational medicine) is responsible for determining whether a worker is fit to work in the MRI environment. Similar procedures take place in other countries. Regarding workers' safety, standard IEC 60601-2-33 (IEC 2022) assumes that if the MRI procedure is performed safely for the patient, it will likewise be safe for the workers, who typically undergo lower levels of exposure. Nevertheless, this standard warns that the presence of metallic medical implants can increase the risk.

Over the years, alongside the increase in MRI examinations (and, correspondingly, in workers involved in MRI), the prevalence of orthopedic prostheses has increased. This increase can be observed not only among patients but also among healthcare staff, especially concerning fracture fixation devices (Bozic et al. 2015; Younger Adults Getting Hip Replacements 2025). The result is a noteworthy chance of finding MRI workers carrying prostheses. Although, in many cases, common sense reasoning suggests that the incremental risk for a worker with a nonferromagnetic orthopedic metallic prosthesis is negligible, the competent authority faces the formal need to certify fitness for the job. From a legal standpoint, it is therefore essential to rely on rigorous scientific sources.

This paper aims to provide quantitative information that can assist the relevant authority in assessing work suitability for workers carrying orthopedic prostheses in presence of the most widespread MRI scanners. To this end, it is assumed that the following conditions are met. First, the analysis is limited to passive implants, with special attention to bulky orthopedic prostheses. Active implants and their leads are not covered. The survey concentrates on metallic implants, which cannot be automatically labeled as “MR safe” (ASTM 2023), but it is assumed that such implants are not ferromagnetic, as this may pose a substantial contraindication to entering the examination room. Second, the assessment is carried out for employees who may stand next to the gantry, but do not enter the bore during scanning. The only hazard that is evaluated for a worker partially entering the bore is the *Lenz effect* (see below), which can occur even if scanning is not in progress. Finally, the entire investigation focuses on the incremental risk associated with the presence of the considered implants. The effects that could take place regardless of the presence of such implants (eg sensory effects due to the motion of the workers through the stray stationary magnetic field, which can be mitigated by reducing the speed of motion (Zilberti et al. 2016a)) are not considered. The incremental risk is investigated with reference to the 3 fields produced by the MRI scanner, namely:

- The radiofrequency (RF) field, which can induce heating in biological tissues by thermal power deposition. The presence of a metallic implant may concentrate the thermal power in certain regions causing *hot spots* (Winter et al. 2021). Exposure to the RF field occurs only if scanning is in progress.
- The switched gradient magnetic field, which can induce eddy currents in the metallic implants. Such currents release thermal power, which diffuses toward the surrounding tissues (Winter et al. 2021). Moreover, the gradient magnetic field induces electric fields in the tissues, which can trigger peripheral nerve stimulation (PNS). The presence of an implant may modify the magnitude and spatial distribution of this electric field (Zilberti et al. 2024), possibly reducing the PNS threshold (Yang et al. 2025). Gradient-induced effects can only occur during scanning.
- The stationary magnetic field, which in most MRI settings is permanently on. Motion of a metallic implant through this field induces eddy currents in the implant itself. The interaction between such currents and the stationary magnetic field gives rise to a mechanical force (the Lorentz force), and associated torques. These mechanical actions counteract the change of magnetic flux experienced by the metallic object moving through the magnetic field. This interaction is known as the Lenz effect (ACR Committee on MR Safety 2026).

## Methods

An experimental investigation of the physical effects induced by an MRI scanner in workers' bodies would require complex invasive measurements. Thus, the entire investigation is performed through numerical simulations. Two realistic human models belonging to the virtual population (Gosselin et al. 2014) are used: YoonSun (female, height: 1.52 m, weight: 54.6 kg, BMI: 23.6 kg m^−2^) and Glenn (male, height: 1.73 m, weight: 61.1 kg, BMI: 20.4 kg m^−2^). Modified versions of these human models have been prepared through a realistic *virtual surgery*, to implant a shoulder prosthesis in YoonSun's right shoulder and a hip prosthesis in Glenn's left hip (Arduino et al. 2022). Both implants are made of a nonferromagnetic CoCrMo alloy (electrical conductivity: 1.16 MS m^−1^; thermal conductivity: 14 W m^−1^ K^−1^; specific heat capacity: 450 J kg^−1^ K^−1^; density: 8445 kg m^−3^). We notice that this alloy is not as common as titanium-based alloys; however, it is adopted here because it maximizes gradient-induced effects. Dielectric and thermal properties of biological tissues, including blood perfusion, are set according to the database of the IT’IS Foundation (IT’IS Database 2025). Both versions of the human models, without and with the prostheses, are discretized into voxels, with 2 mm isotropic resolution, which is adopted as a tradeoff between accuracy and computational burden. In the electromagnetic simulations of gradient-induced heating (in which only the implants are simulated) the resolution is changed to 1 mm. All simulations are carried out with custom tools, developed and validated beforehand (Bottauscio et al. 2014; Bottauscio et al. 2015a, 2015b; Zilberti et al. 2016b, 2025; Arduino et al. 2017, 2026; Zanovello et al. 2026b). To generate the fields, realistic coils are considered, suitable for a cylindrical scanner with the longitudinal axis at 1 m from the floor.

### Radiofrequency field

**Figure 1:**
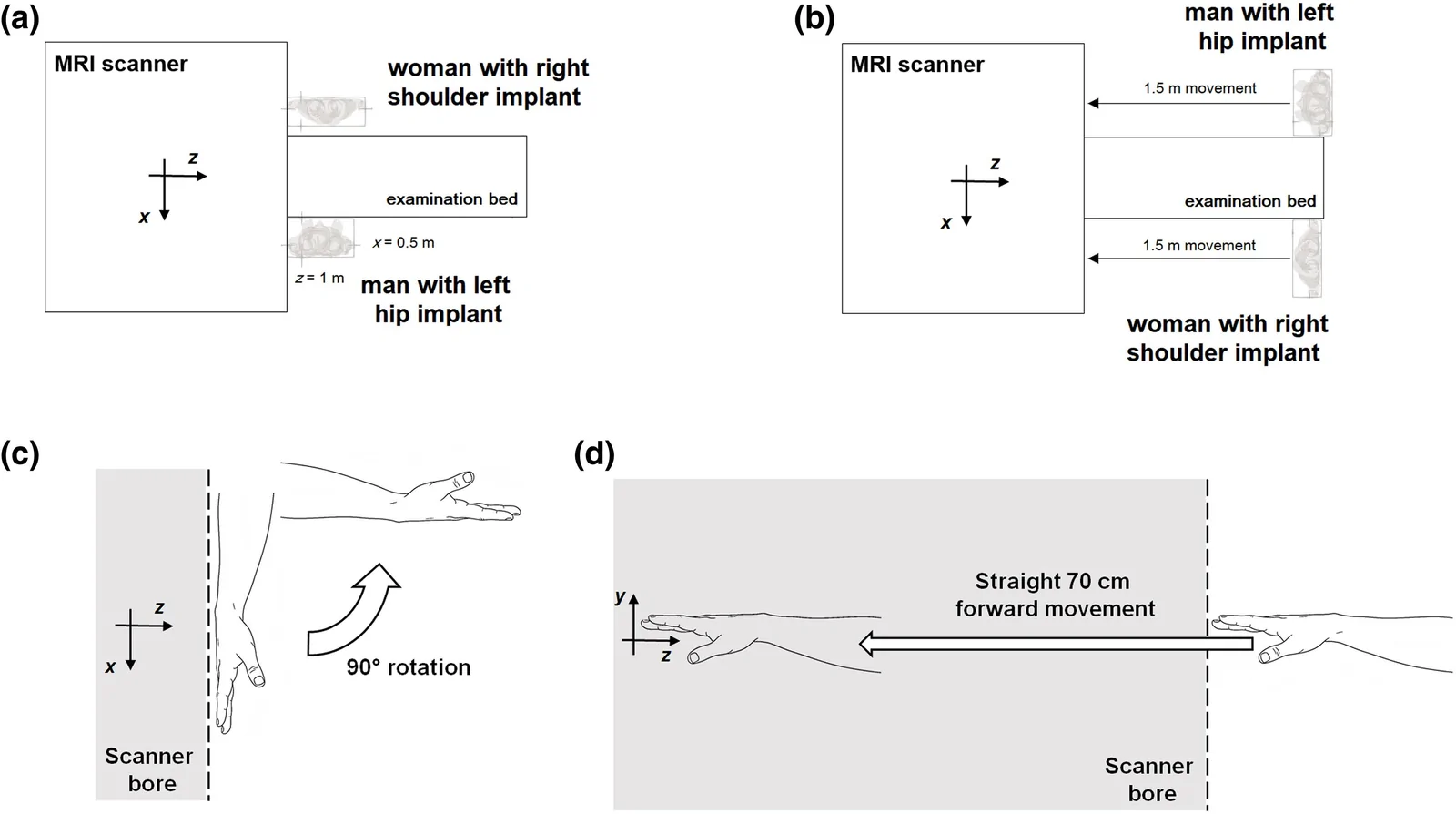
Positions of the two human models considered in the simulations of the exposure to the time-varying fields (a); scheme of the 1.5 m motion of the human models next to the examination bed, performed at a speed of 5 m/s (b); scheme of the 90° rotation of a forearm in front of the scanner bore, performed in 0.4 s (c); scheme of the motion of a forearm which enters the scanner bore by 70 cm in 0.5 s (d).

The exposure to the RF field is investigated with the two human models positioned in the proximity of the scanner entrance, facing the examination bed, with the side of the body featuring the prosthesis close to the bore (Fig. 1a). The incremental risk associated with the presence of the implants is evaluated by comparing the heating produced in the human models with and without the prostheses. The RF field is generated by a 16-leg birdcage body-coil (diameter: 70 cm; axial length: 44 cm) working in CP mode. The power released by the coil is set in such a way to produce, continuously, a whole-body SAR equal to 2 W/kg (ie the limit recommended by the IEC standard (IEC 2022) for the normal operating mode) in the Glenn human model (without implant) placed within the scanner at spine landmark. The simulation is performed twice, at the frequencies of 64 MHz and 128 MHz (relevant to 1.5 T and 3 T scanners, respectively). With the considered RF coil, the adopted exposure condition results in a *B*_1_^+^ of 14.6 μT at 64 MHz and 7.50 μT at 128 MHz, at the coil center. Heating is calculated using Pennes' bioheat equation with constant parameters (Arduino et al. 2017). To obtain a conservative estimate of the heating, the birdcage is operated continuously for 30 min.

### Switched gradient magnetic field

To assess the exposure to the gradient field, the two human models are positioned again as in Fig. 1a. The gradient magnetic field is generated by coils with sensitivity ∼57 μT m^−1^ A^−1^. To evaluate gradient-induced heating of the implants, two imaging sequences are considered: a balanced steady state free precession (bSSFP) sequence with maximum slew rate of 200 T m^−1^ s^−1^, and an echo planar imaging (EPI) sequence with maximum slew rate of 167 T m^−1^ s^−1^. The complete details of these sequences are reported in a previous paper (Arduino et al. 2021), where they were identified as the sequences with the largest potential for gradient-induced heating in patients with implants. The simulation of the EPI sequence is performed three times, each time placing the readout signal (which provides the main heating contribution) on a different gradient axis. Tissue heating is computed by simulating the diffusion of the thermal power released by eddy currents inside the implants. To perform a conservative assessment, a 30 min exposure at a 100 % duty cycle is simulated. Since gradient fields cannot produce direct heating of biological tissues, the evaluation of the incremental risk due to the introduction of the prostheses in the workers' bodies does not require a comparison to the scenario where the prostheses are absent.

Another experiment, designed to check if the presence of the prostheses can boost the likelihood of PNS, is performed feeding the gradient axes alternately, with a sinusoidal current. In this case, the simulations provide the magnitude of the electric field induced in the body, both when the implants are present and when they are absent. To have a further term of comparison, the same calculation is performed in the body of a patient without implants, who undergoes abdominal imaging in a supine position (Glenn model is used). To mitigate the presence of numerical outliers in the results, the raw data produced by the simulations are post-processed through a suitable statistical filter (Arduino et al. 2020).

### Stationary magnetic field

The Lenz effect is studied with reference to the trajectories depicted in Fig. 1b, where the two human models move toward the scanner, covering a distance of 1.5 m. For both models, the side of the body with the prosthesis is kept close to the examination bed. The speed of motion is set at a high value of 5 m s^−1^, to represent the reaction to an emergency. The investigation of the Lenz effect is broadened to include a forearm fixation plate (length: 89 mm; width: 23 mm; thickness: 3.2 mm) made of a nonferromagnetic Ti6Al4 V alloy (electrical conductivity: 0.58 MS m^−1^; density: 4420 kg m^−3^). This implant is discretized into voxels with isotropic resolution of 0.4 mm. In the first instance, the plate is positioned in front of the scanner entrance, with the largest surface orthogonal to the *Z*-axis. To simulate the abrupt rotation of a worker's forearm (Fig. 1c), the plate is rotated 90° around a vertical axis, in 0.4 s. In another experiment, the plate is placed horizontally at the bore entrance, parallel to the scanner axis. To simulate the movement of a forearm that quickly enters the bore (Fig. 1d), the plate moves by 70 cm in 0.5 s, toward the scanner isocenter. This is the only instance in which a prosthesis is simulated inside the scanner. In all tests, a 3 T scanner is considered. The distribution of the stationary magnetic field is generated by a set of coils with active shielding. The forces and torques produced on the implants due to the Lenz effect are compared to the weight and the worst case gravitational torque (WCGT) of the implants themselves. The WCGT is defined as the product of the weight and the maximum linear length of the implant (ASTM 2017).

## Results

### Radiofrequency field

**Figure 2:**
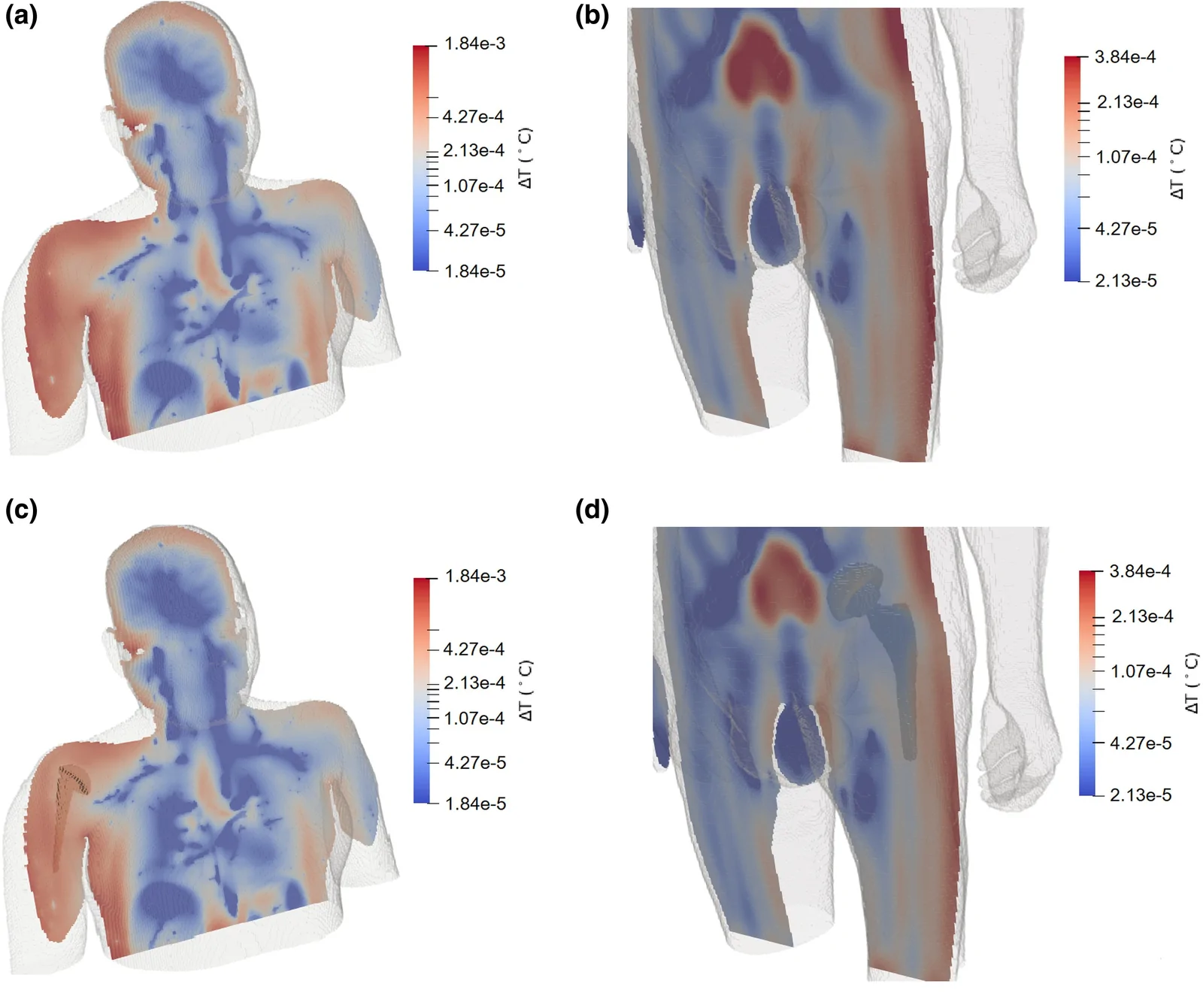
Temperature increase (°C) induced by the RF field at 64 MHz on a coronal cross section: female worker without implants (a); male worker without implants (b); female worker with right shoulder implant (c); male worker with left hip implant (d).

The magnitude of the RF magnetic induction produced by the birdcage coil in the left hip of the male worker and in the right shoulder of the female worker (placed as in Fig. 1a) roughly amounts to 0.10 and 0.15 μT, respectively, at 64 MHz, and to 0.052 and 0.075 μT, respectively, at 128 MHz, when the prostheses are not present. These values are about 2 orders of magnitude lower than the value at the center of the birdcage. The maximum values of the temperature increase observed in the bodies of the two workers, without and with the implants, are reported in Table 1. An example of the spatial distribution of the temperature increase induced by the RF field at 64 MHz is given in Fig. 2.

**Table 1:** Maximum temperature increase (°C) induced in the body of the workers due to the exposure to time-varying fields.

Human model		RF field		Gradient field
		64 MHz	128 MHz	
Female worker	Without implant	2.3 × 10^−3^	2.9 × 10^−3^	n/a
	With implant	4.5 × 10^−3^	2.8 × 10^−3^	5.2 × 10^−5^ (bSSFP) 4.6 × 10^−6^ (EPI)
Male worker	Without implant	6.6 × 10^−4^	7.9 × 10^−4^	n/a
	With implant	5.1 × 10^−4^	7.3 × 10^−4^	3.6 × 10^−4^ (bSSFP) 2.5 × 10^−5^ (EPI)

For the EPI sequence, the reported value refers to the worst case observed by feeding either the *X*-, or *Y*-, or *Z*-axis of the gradient coils with the readout signal. For both RF and gradient fields, a continuous 30-min exposure has been simulated.

### Switched gradient magnetic field

**Figure 3:**
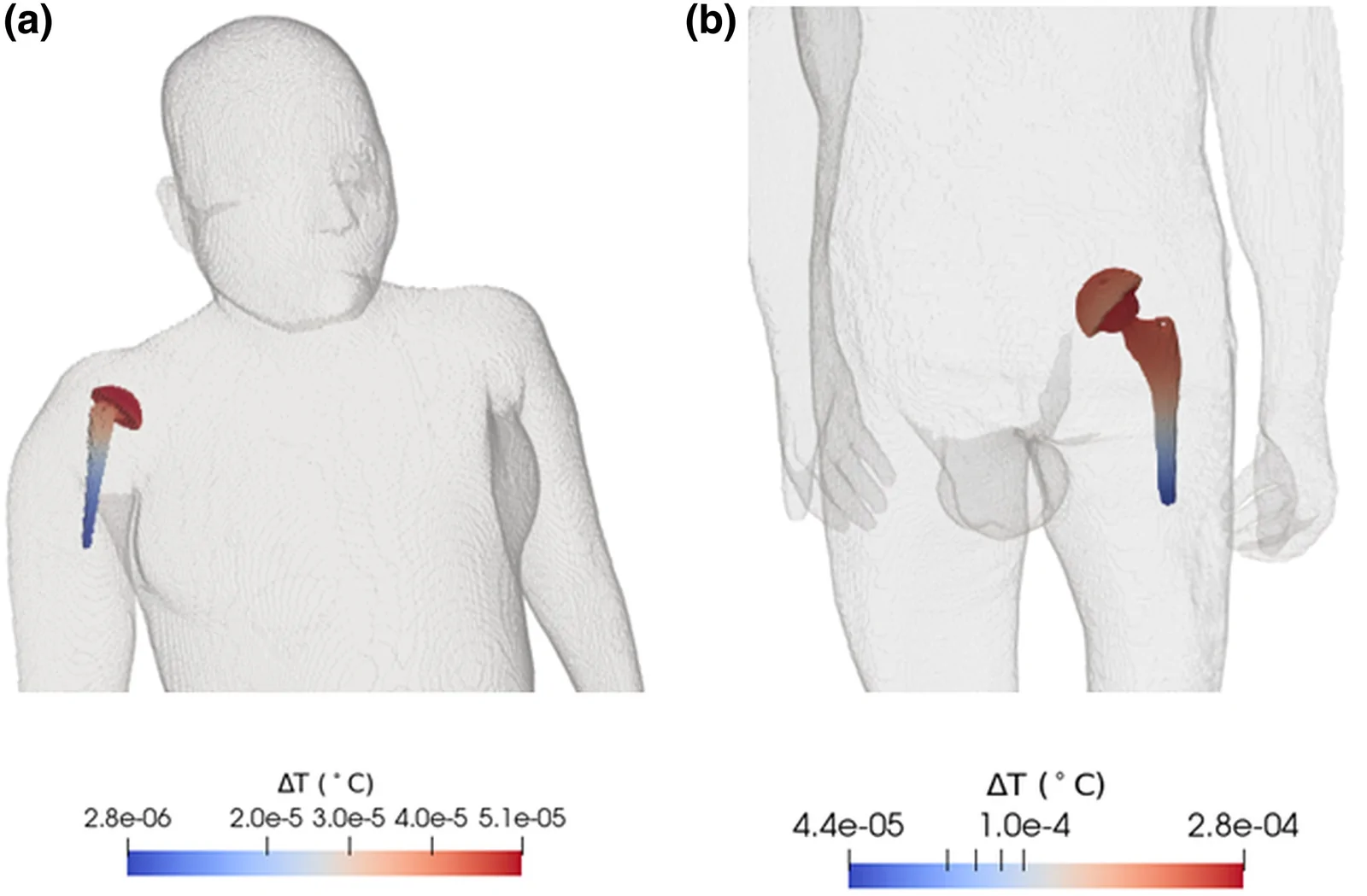
Temperature increase (°C) induced by the bSSFP gradient sequence on the external surface of the shoulder prosthesis implanted in the right side of the female model (a) and on the external surface of the hip prosthesis implanted in the left side of the male model (b).

The level of exposure to gradient fields depends on the applied imaging sequence. When feeding one axis of the gradient coils at a time, the spatial peak of the magnetic induction in the bodies of the workers is at least 60 times smaller than the peak observed in the body of a patient who lies inside the scanner at the abdominal landmark. This means that, if such a patient is exposed to a maximum time-derivative of magnetic induction equal to 42 T/s (ie the value recommended in ISO/TS 10974 (ISO 2018) to test gradient-induced heating of active implants), the exposure of the considered workers at the worst location is 0.7 T/s. Maximum values of the temperature increase induced in the workers' bodies by the bSSFP and EPI sequences are reported in Table 1. The spatial distribution of the temperature increase on the external surface of the implants is displayed in Fig. 3 for the bSSFP sequence.

The results of the simulations performed to investigate possible effects on PNS are summarized in Table 2, where the peak magnitude of the electric field induced in the bodies of the workers is reported as a percentage of the peak magnitude obtained in the patient's body. The analysis is performed considering either all tissues or focusing on the nerves only.

**Table 2:** Peak value of the electric field induced in the body of the workers, expressed as a percentage of the peak value in the body of the patient.

		Female worker		Male worker	
		All tissues	Nerves only	All tissues	Nerves only
*X*-axis	Without implant	13 %	6.4 %	9.8 %	4.6 %
	With implant	12 %	6.3 %	24 %	5.7 %
*Y*-axis	Without implant	1.7 %	1.5 %	2.0 %	1.1 %
	With implant	3.5 %	2.6 %	5.9 %	2.0 %
*Z*-axis	Without implant	2.9 %	2.4 %	2.1 %	1.9 %
	With implant	3.6 %	2.7 %	6.7 %	2.8 %

### Stationary magnetic field

**Figure 4:**
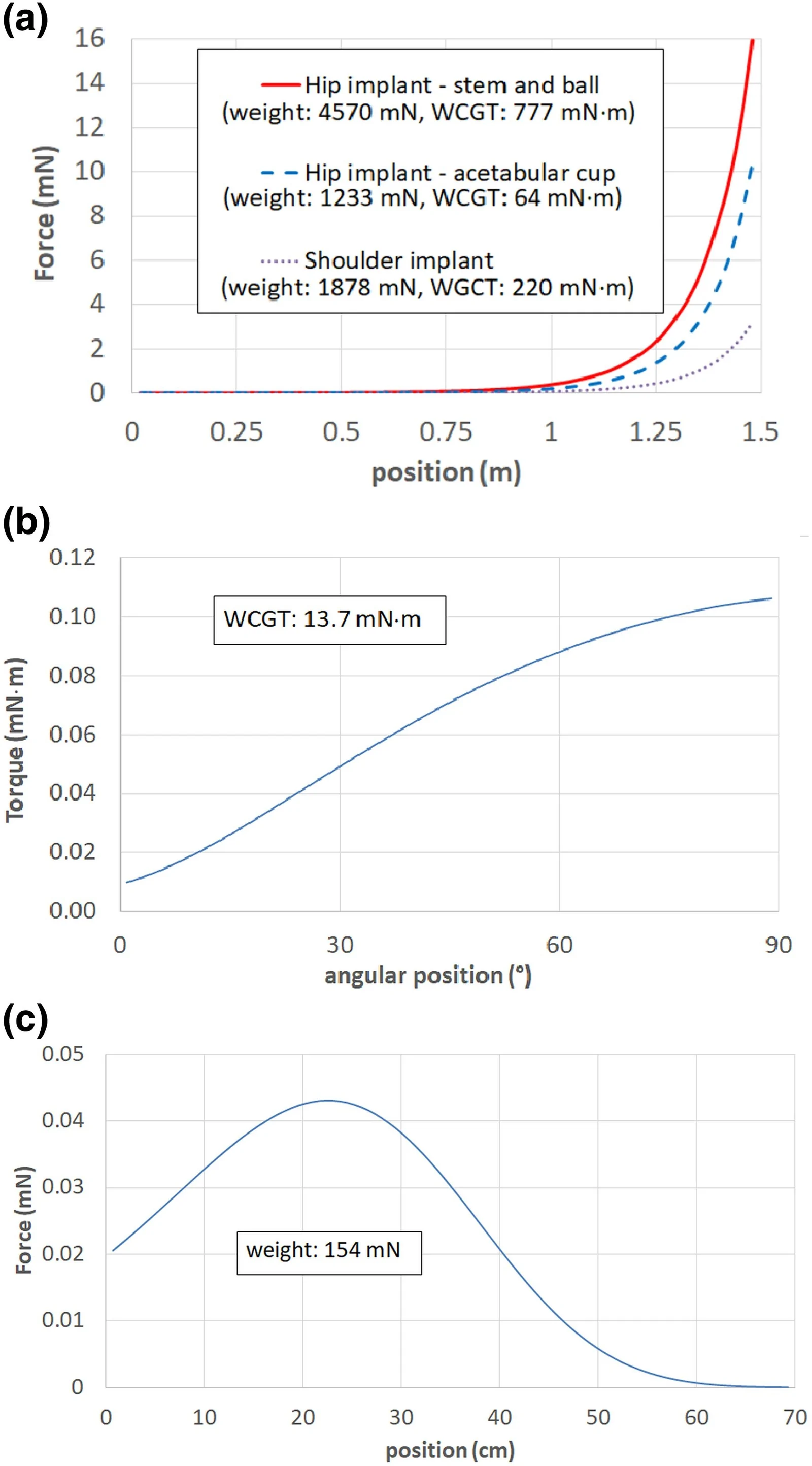
Lenz effect on the considered implants. Magnitude of the force (mN) exerted on the prostheses implanted in the body of the two workers, expressed as a function of the position along the trajectories indicated in Fig. 1b (a). Torque (mN m) acting on the forearm plate during the rotation depicted in Fig. 1c (b). Force (mN) acting on the forearm plate during the translation depicted in Fig. 1d (c). The weight and the worst case gravitational torque (WCGT) of the implants are indicated in the insets.

The magnitude of the forces exerted on the implants of the two workers moving next to the examination bed (Fig. 1b) is reported in Fig. 4a as a function of the position along the trajectory. Such forces are far smaller than the weight of the implants. Similarly, the torques on the implants are much lower than their WCGT (the highest value is obtained for the acetabular cup of the hip prosthesis implanted in the body of the male worker, which amounts to about 1.7 mN m).

The results of the simulations of the movements indicated in Fig. 1c and d are summarized in Fig. 4b and c, respectively. The former shows the magnitude of the torque that tries to counteract the rotation of the forearm plate in front of the bore; the latter shows the magnitude of the force that opposes the linear motion of the plate toward the center of the scanner. Also in these cases, the magnitude of the mechanical actions due to the Lenz effect is orders of magnitude lower than the WCGT and the weight of the implant. The same applies to the net force acting during the rotation (max: 0.282 mN) and to the torque acting during the translation (max: 0.00204 mN m).

## Discussion

At radiofrequencies, the presence of prostheses in the bodies of the two workers does not produce significant overheating (actually, in some instances, the presence of the bulky metallic objects makes the maximum temperature lower than that achieved without the implants). Specifically, highest temperature rise remains on the order of 5 × 10^−3^ °C when scanning the patient at whole-body SAR equal to 2 W/kg. Assuming linearity (which is justified due to the small level of heating, which should not trigger thermoregulation), the highest temperature rise in the workers' bodies would scale linearly with respect to the SAR in the patient's body, and quadratically with respect to *B*_1_^+^ generated in the scanner center. Hence, the maximum temperature increase would reach about 0.01 °C working at whole-body SAR equal to 4 W/kg, which is the limit recommended for the first level controlled operating mode (IEC 2022). Gradient-induced heating is even smaller, despite the use of the highly conductive CoCrMo alloy. Both RF- and gradient-induced heating depend on the considered implants and imaging sequences (information about the variability of these types of heating can be found in (Wooldridge et al. 2021)), but the values obtained here are so small to allow inferring that a wide safety margin exists. We notice that, in the considered scenarios, RF-induced heating is worse for the female worker, whereas gradient-induced heating is worse for the male worker. However, this result stems from multiple factors (including model anatomy, position of the implants with respect to the scanner axis, type and size of the prostheses) and therefore it should not be taken as a general rule. We also notice that, during an imaging sequence, exposure to the RF and gradient fields occurs simultaneously; hence, these two sources could have an additive effect (Zanovello et al. 2026a). Even if this were to happen, the induced temperature increase would stay below a hundredth of a degree Celsius, in spite of the considered strenuous conditions (30 min of continuous exposure). This indicates that the heating due to the presence of the considered implants should never constitute a risk factor in the investigated situations. Extension of this result to other types of passive implants requires some caution. However, the available elements allow drawing some general advice. The RF and gradient fields outside the scanner exhibit the same waveforms as the fields inside the scanner. In addition, both RF- and gradient-induced heating effects scale with the square of the magnitude of the fields. Previous investigations (Zanovello et al. 2026a) indicate that during scanning inside conventional cylindrical scanners, other passive implants, like long ankle plates and large cranial plates, typically produce a heating lower than that produced by joint replacements (which can amount to some degrees Celsius, under conditions similar to those considered here). Our simulations indicate that the workers standing outside the scanner are exposed to field magnitudes that are over one order of magnitude less than the corresponding magnitudes inside the scanner. Due to the different spatial distribution of the fields inside and outside the scanner, data observed within the scanner cannot straightforwardly be adjusted for implants located outside the scanner. Nonetheless, all things considered, a heating lower than 5 × 10^−3^ °C is expected in the bodies of workers carrying such other implants and standing outside a scanner running in normal operating mode.

Besides heating, gradient-induced eddy currents may also produce, in the presence of a stationary magnetic field, mechanical vibrations of metallic objects. The extent of such vibrations was previously studied for an MRI patient with orthopedic prostheses and found to be moderate (Zilberti et al. 2025). In the light of the observed exposure levels, this suggests that gradient-induced vibrations of orthopedic prostheses do not represent a sensible source of risk for a worker standing next to the scanner.

Regarding the magnitude of the electric field induced by gradient fields in biological tissues, the introduction of the prostheses into the workers' bodies generally produces an amplification (an exception is found for the female worker with shoulder implant, exposed to the field produced by the *X*-axis of the gradient coil system). This effect is found in the nerves of the human models and also in all other tissues, where smaller nerves, not represented within the adopted anatomical models, may be present. However, this electric field is always weaker than the field induced in the patient's body, and, on the whole, is still comparable to the field observed in the models of the workers without the implants. We remark that PNS is a complex phenomenon, which depends on the coupling between the electric field and the nerve network (Davids et al. 2019). Therefore, the accurate prediction of the PNS threshold in the workers' bodies would require a neurodynamic simulation, and the use of the electric field as a metric to evaluate the likelihood of PNS must be seen as a surrogate for more advanced investigations. On the other hand, standard IEC 60601-2-33 (IEC 2022) itself provides PNS thresholds in terms of the magnitude of the induced electric field (or, alternatively, of the time-derivative of the applied magnetic induction). Thus, even if this approach suffers from certain limitations, it is adopted here to derive conclusions, meant in a probabilistic manner. Specifically, the results suggest that the workers benefit from a good safety margin, provided they stay outside the scanner and no PNS is induced in the patient's body. Due to the linearity of the problem, this remains valid regardless of the frequency and magnitude of the applied gradient field. In the event that a moderate PNS manifests in the workers' bodies, they should be able, thanks to the training received, to recognize and manage the discomfort that may arise, without it leading to unacceptable risk situations.

Regarding the Lenz effect, notwithstanding the adopted high-speed values, the forces and the torques on the implants are always much smaller than the corresponding weight and WCGT. This occurs even if the implant is close to, or enters, the scanner bore. In terms of force, the worst case is observed for the stem and ball of the hip prosthesis implanted in the body of the male worker. In this case, the maximum force amounts to about 16 mN, which is roughly equivalent to the weight of a 1.6 g mass. Since the considered hip prosthesis is relatively large, it is possible to infer that the Lenz effect occurring in other kinds of orthopedic implants, with small cross section (eg nails) or thin thickness (eg plates), is weaker, in general. This result suggests that the worker is not even able to perceive this effect, which therefore does not introduce an element of additional risk. We notice that, for a given trajectory and speed of motion, the magnitude of the mechanical actions due to the Lenz effect depends on the spatial gradient of the magnetic field experienced by the moving implant (which determines the strength of the currents induced in the implant) and on the magnitude of the magnetic field itself. Thus, the forces and torques calculated for the 3 T scanner would decline to 25% if the scanner produced a stationary magnetic field with exactly the same spatial distribution as simulated here but at half the magnitude (hence, 1.5 T at the isocenter). Actually, the spatial pattern of the stationary magnetic field may change based on the scanner design. Therefore, the accurate quantification of the Lenz effect occurring with other scanner designs would require specific knowledge of the stray field produced by the considered scanners.

## Conclusions

The analysis indicates that the additional risk associated with the presence of a metallic orthopedic prosthesis implanted in the bodies of personnel working around an MRI scanner is minimal. This finding aligns with the empirical evidence that, in many cases, the risk associated with the presence of a passive implant during an MRI procedure is low even for the patients (Ashmore et al. 2025). This conclusion has been reached by analyzing a specific set of sources, producing specific spatial distributions of RF, gradient and stationary magnetic fields. Nevertheless, the low extent of the physical effects induced by these sources suggests that a good safety margin exists, such that the final outcomes of the analysis can be extended to the most common MRI environments. Apart from this aspect, the limitations of this investigation closely correspond to the assumptions under which the simulations were conducted, which can be summarized in 3 main restrictions. First, the analysis does not cover active implants, which could introduce other elements of risk. Second, the investigation excludes the presence of ferromagnetic components in the prostheses. Such a presence (unlikely to occur) would require a dedicated risk assessment. Finally, the analysis focuses on the exposure of workers placed outside the scanner. The only considered exception is the Lenz effect on a forearm plate (which may take place, eg when a worker cleans the inner volume of the scanner). If workers with prostheses enter the scanner during imaging (eg in interventional MRI), their exposure becomes comparable to that of patients with implants, for whom extensive literature is available (see, eg Powell et al. 2012; Arduino et al. 2019, 2021; Davids et al. 2019; Winter et al. 2021; Wooldridge et al. 2021; Clementi et al. 2022; Espiritu et al. 2023; Shellock et al. 2024; Yang et al. 2024, 2025; Zilberti et al. 2024, 2025; Ashmore et al. 2025; Zulkarnain et al. 2025; Akter et al. 2025a, 2025b; Zanovello et al. 2026a, 2006b). Currently, the occurrence of this situation can still be considered rare and beyond the scope of this paper. As a final remark, we also remind that this analysis has been conceived to quantify the additional risk due to the presence of orthopedic prostheses. The evaluation of the baseline risk, common to any worker, may require assessing other safety aspects (see, for instance, Bravo et al. 2021), which are taken for granted here.

## Data Availability

The data underlying this article will be shared on reasonable request to the corresponding author.
